# SNS-032 attenuates liver fibrosis by anti-active hepatic stellate cells *via* inhibition of cyclin dependent kinase 9

**DOI:** 10.3389/fphar.2022.1016552

**Published:** 2022-10-12

**Authors:** Xiao-Li He, Yong-Hong Hu, Jia-Mei Chen, Ding-Qi Zhang, Hai-Lin Yang, Lin-Zhang Zhang, Yong-Ping Mu, Hua Zhang, Gao-Feng Chen, Wei Liu, Ping Liu

**Affiliations:** ^1^ Key Laboratory of Liver and Kidney Diseases (Ministry of Education), Institute of Liver Diseases, Shanghai Key Laboratory of Traditional Chinese Clinical Medicine, Shuguang Hospital Affiliated to Shanghai University of Traditional Chinese Medicine, Shanghai, China; ^2^ Department of Endocrinology, Yueyang Hospital of Integrated Traditional Chinese and Western Medicine, Shanghai University of Traditional Chinese Medicine, Shanghai, China; ^3^ Shanghai Frontiers Science Center of TCM Chemical Biology, Institute of Interdisciplinary Integrative Medicine Research, Shanghai University of Traditional Chinese Medicine, Shanghai, China

**Keywords:** liver fibrosis, hepatic stellate cell, cyclin-dependent kinase 9, apoptosis, activation, proliferation

## Abstract

Liver fibrosis is a common pathological process of all chronic liver diseases. Hepatic stellate cells (HSCs) play a central role in the development of liver fibrosis. Cyclin-dependent kinase 9 (CDK9) is a cell cycle kinase that regulates mRNA transcription and elongation. A CDK9 inhibitor SNS-032 has been reported to have good effects in anti-tumor. However, the role of SNS-032 in the development of liver fibrosis is unclear. In this study, SNS-032 was found to alleviate hepatic fibrosis by inhibiting the activation and inducing the apoptosis of active HSCs in carbon tetrachloride-induced model mice. *In vitro*, SNS-032 inhibited the activation and proliferation of active HSCs and induced the apoptosis of active HSCs by downregulating the expression of CDK9 and its downstream signal transductors, such phosphorylated RNA polymerase II and Bcl-2. CDK9 short hairpin RNA was transfected into active HSCs to further elucidate the mechanism of the above effects. Similar results were observed in active HSCs after CDK9 knockdown. In active HSCs with CDK9 knockdown, the expression levels of CDK9, phosphorylated RNA polymerase II, XIAP, Bcl-2, Mcl-1, and ɑ-SMA significantly decreased, whereas those of cleaved-PARP1 and Bax decreased prominently. These results indicated that SNS-032 is a potential drug and CDK9 might be a new prospective target for the treatment of liver fibrosis.

## Introduction

Liver fibrosis is a general pathological change that is induced by several chronic liver diseases, such as viral hepatitis, nonalcoholic steatohepatitis, primary cholestatic cirrhosis, and drug-induced liver disease. It is characterized by the excess accumulation of the extracellular matrix (ECM) in liver tissue (Strowitzki et al., 2019). The core event of fibrosis is the activation of hepatic stellate cells (HSCs), which synthesize and secrete large amounts of ECM (Tanwar et al., 2020). Acta2 (α-smooth muscle actin, α-SMA), the marker of active HSCs, and collagen type Ⅰα1 (Col1A1), one of the most crucial ECM proteins, are usually detected to evaluate the activation of HSCs and the stage of fibrosis (Chen et al., 2010a; Jin et al., 2011). When fibrosis develops, the production and apoptosis of active HSCs increase, with production exceeding apoptosis. This condition leads to the increase in the total number of active HSCs. In the recovery stage of fibrosis, the opposite phenomenon occurs, and the number of active HSCs decreases. Recent studies have demonstrated that the apoptosis of active HSCs could be induced by aquaporins and cytokines, such as nerve growth factors, and eliminated by natural killer cells (Oakley et al., 2003; Lakner et al., 2011; Kisseleva and Brenner, 2012). Although the apoptosis of active HSCs was observed many years ago, methods for selectively inducing this process remain lacking.

Cyclin-dependent kinase 9 (CDK9) is a member of the cyclin-dependent kinase (CDK) family (Matrone et al., 2015). Recent studies have suggested that CDKs play an important role in cell apoptosis, proliferation, activation, and survival (Santo et al., 2015). The CDK subfamily members CDK2, CDK4, and CDK6 regulate the cell cycle, whereas other CDK subfamily members, such as CDK7 and CDK9, affect the transcription of mRNA (Gérard and Goldbeter, 2016). CDK9 binds with cyclin families (cyclin T1, T2a, T2b, or K) to form a heterodimer kinase called positive transcription elongation factor b (P-TEFb), which is necessary and crucial for promoting the transcription and elongation of mRNA by activating RNA polymerase II (RNAP II) (Fujinaga, 2020). P-TEFb has two states: inactive and catalytically active. Commonly, P-TEFb is inactive when it is bound with hexamethylene bis-acetamide-inducible protein 1 (HEXIM1), methylphosphate capping enzyme (MePCE), Larp7, and 7SK small nuclear ribonucleoprotein (7SK snRNP) (Fujinaga et al., 2014). When DNA damage is induced by ultraviolet rays or other physical and chemical injuries, inactive P-TEFb releases HEXIM1, MePCE, Larp7, and 7SK snRNP and recruits bromodomain-containing protein 4 (BRD4) (or super elongation complex [SEC]; whichever factor is recruited varies in different cells), which activates the phosphorylation of CDK9 and provides the catalytic activity that can phosphorylate RNAP II (Zheng et al., 2021). RNAP II is one of the most important synthetic enzymes of mRNA elongation in eukaryotes (Liang et al., 2015). Phosphorylated CDK9 is the core of active P-TEFb, which finally determines the catalytic activity of P-TEFb. CDK9 inhibitors can inhibit the phosphorylation of RNAP II *via* inhibiting the phosphorylation of CDK9 and degrading CDK9 to block the activation of P-TEFb (Bose et al., 2013; Zhou et al., 2022).

SNS-032, a CDK9 inhibitor, has the IC_50_ ratio of inhibition with CDK9 of 4 nM, which indicates 10-fold selectivity over other CDKs (48 nM to CDK2, 62 nM to CDK7, 340 nM to CDK5, and little inhibitory effect on CDK6) *in vitro* (Chen et al., 2009; Conroy et al., 2009). The safety of SNS-032 has been evaluated in phase I clinical trial. And it has been used to treat hematopoietic diseases, such as B-lymphoid malignancies, chronic lymphocytic leukemia, mantle cell lymphoma, and multiple myeloma (NCT00446342), as well as some advanced solid tumors (NCT00292864) (Tong et al., 2010; Walsby et al., 2011). Recently, it has also been used to treat solid tumors, such as ovarian carcinoma, by preventing the proliferation and formation of capillaries through the inhibition of vascular endothelial growth factors (Misra et al., 2004; Ali et al., 2007). A recent study has shown that knockdown of CDK9 and a CDK9 inhibitor (LDC000067) could remarkably ameliorate renal fibrosis by blocking the phosphorylation of the Smad3 linker (Thr179) (Qu et al., 2015). However, the role of CDK9 inhibitors in the development of liver fibrosis is unclear. Therefore, the purpose of this study is to clarify the antiliver fibrosis effects of the CDK9 inhibitor SNS-032 and elucidate its possible mechanism *in vivo* and *in vitro*.

## Materials and methods

### Drugs and reagents

SNS-032 (lot CSN12378, a specific CDK9 inhibitor) was purchased from CSNpharm, Inc. (Shanghai, China). SB431542 [lot S1067, a transforming growth factor-β [TGF-β] receptor inhibitor and used as the positive drug for inhibiting activated HSCs (Gupta et al., 2016)], was obtained from Selleckchem, Inc. (Shanghai, China). Sorafenib tosylate, which was used as the positive drug *in vivo* as previously described (Lin et al., 2016), was bought from ChemBest Research Laboratories Ltd. (Shanghai, China). CCl_4_ was acquired from China Pharmaceutical Group Co., Ltd. (Beijing, China). Fetal bovine serum (FBS), Dulbecco modified Eagle’s medium (DMEM), penicillin–streptomycin solution, 0.25% trypsin with ethylenediamine tetra-acetic acid (EDTA), and 0.25% trypsin without EDTA were bought from Gibco (Thermo Fisher Scientific, Inc., MA, United States). Dimethyl sulfoxide was purchased from Sigma-Aldrich, LLC. (MUC, GER). Phosphate-buffered saline (PBS) was procured from Hyclone (Thermo Fisher Scientific, Inc., MA, United States). Recombinant human TGF-β1 was obtained from R&D (R&D Systems Inc., MN, United States). Serum alanine aminotransferase (ALT), aspartate transaminase (AST), Sirius red staining kit, and hematoxylin–eosin (HE) staining kit were provided by Jiancheng Bioengineering Institute (Nanjing, China). Annexin V–fluorescein isothiocyanate (FITC)/propidium iodide (PI) apoptosis kit and annexin V–allophycocyanin (APC) dye were purchased from BD Biosciences, Inc. (NJ, United States). Diaminyl phenyl indole (DAPI) dye was acquired from Beyotime Biotechnology, Inc. (Shanghai, China).

### Animals and treatment

Forty-eight male C57BL6 mice (6-week-old, 18–20 g) were caged individually in a temperature- and humidity-controlled environment under a 12:12 light–dark cycle. After acclimation, they were kept in the Shanghai Model Organisms Center, Inc. (Shanghai, China) with the license number SCXK 2019-0002. Then, they were assigned into two groups randomly. The first group (control group, *n* = 8) was fed with normal diet and water freely. The second group (*n* = 40) was intraperitoneally injected with 2 ml·kg^−1^ 15% carbon tetrachloride (CCl_4_)–olive oil on Mondays, Wednesdays, and Fridays for 3 weeks. Then, the mice in the second group were randomly allocated into five groups (experimental, low-dose SNS-032, medium-dose SNS-032, high-dose SNS-032, and sorafenib groups; *n* = 8). Subsequently, the mice in the low-, medium-, and high-dose SNS-032 and sorafenib groups were treated with 2.5, 5, and 10 mg·kg^−1^·day SNS-032 through intraperitoneal administration or 5 mg·kg^−1^·day sorafenib through intragastric administration for 3 weeks with the continuous injection of CCl_4_–olive oil. The CCl_4_ mouse model was used as the liver fibrosis model as previously described (Strnad et al., 2008).

All experiments were approved (ID. PZSHUTCM190315006) by the experimental animal ethics committee of the Shanghai University of Traditional Chinese Medicine and conducted in accordance with the Guide for the Care and Use of Laboratory Animals (National Institutes of Health, NIH Publication 86–23, revised 1996).

### Cell culture

The human hepatic stellate cell line LX-2 was obtained from the Institute of Liver Disease affiliated with the Shanghai University of Traditional Chinese Medicine. LX-2 was cultured with DMEM containing 10% FBS in 5% CO_2_ at 37 C. The cells were passaged when their density reached 80%.

### Cell treatment

The human hepatic stellate cell line LX-2 was cultured on six-well plates or 6 cm culture dishes at the density of 2 × 10^6^ cells per well or 4 × 10^6^ cells per dish for 24 h. LX-2 was incubated with 5 ng·mL^−1^ TGF-β1 for 24 h to construct the activated HSC model *in vitro* as previously described (He et al., 2020)*.* Then, the activated LX-2 cell line was treated with 0.04, 0.2, and 1 μM SNS-032 for 24 h H_2_O_2_ with the concentration of 200 μM was used as the positive drug in apoptosis induction (Shang et al., 2020), and 10 μM SB431542 was used as the positive drug in the inhibition of activated LX-2 (Premkumar and Shankar, 2021). Then, the cell lysates of each group were collected and stored at −80 °C in a refrigerator.

### Establishment of the CDK9 knockdown LX-2 cell line

A CDK9 knockdown strategy using lentiviral vectors encoding CDK9 short hairpin RNA (shRNA) was utilized. LX-2 cells were incubated with lentiviral vectors at MOI ratio = 40 for 8 h. Then, the transfected cells were cultured and incubated with 4 μg·mL^−1^ purinomycin for 48 h to screen LX-2 cells with CDK9 knockdown. The lentiviral vectors (GV248) that carried CDK9 shRNA, green fluorescent protein (GFP), and antipurinomycin genes were designed, synthesized, and packed into the GV248 lentivirus by Genechem, Inc. (Shanghai, China). The RNA sequences of the three CDK9 shRNAs are listed in Supplementary Table S1.

### HE staining

Liver tissues were dehydrated in a gradient series of alcohol and embedded in paraffin wax blocks. Before staining, 4 μm-thick liver tissue sections were dewaxed in xylene, rehydrated in decreasing concentrations of ethanol, and washed in PBS. Then, the sections were stained with HE. After staining, the sections were dehydrated in increasing concentrations of ethanol and xylene and covered with neutral resin.

### Sirius red staining

Liver paraffin sections were dewaxed and rehydrated. Next, the slices were incubated with Sirius red dye for 10 min at 37 C. After being washed with ethanol for 2 min twice, the slices were immersed in dimethylbenzene for 10 min and covered with neutral resin and cover slips. High-resolution scanning and photography were performed with a Leica SCN400 Slide Scanning System (Leica, Inc., Wetzlar, GER). The positive area of collagen was semiquantitatively analyzed by using Aperio Scope software (Leica, Inc., Wetzlar, GER).

### Biochemical assays

The serum levels of AST, ALT, TBIL, and ALB were measured with test kits purchased from Jiancheng Bioengineering Institute (Nanjing, China) and a Beckman Coulter Synchron DXC800 Automatic Biochemical Analyzer (CA, United States) as described by the manufacturer.

### Immunohistochemical staining

The liver paraffin sections were dewaxed and rehydrated first. After antigen repair and endogenous peroxidase elimination, the sections were treated with 10% goat serum for 30 min and subsequently covered with 1 μg·mL^−1^ Acta2 primary antibody (see Supplementary Table S2) at 4 C for 12 h. Horseradish peroxidase solution was added to conjugate the primary antibody, and 2% diaminobenzidine solution was used to visualize the positive area. The sections were stained with hematoxylin dye to visualize nuclei. Positive stained areas were semiquantitatively analyzed by using Aperio Scope software (Leica, Inc., Wetzlar, GER).

### Annexin V–FITC/propidium iodide staining

After TGF-β1 and SNS-032 treatment for 24 h, the cells were digested with 0.25% trypsin, washed twice with cold PBS, and resuspended in 1× binding buffer at the concentration of 1 × 10^5^ cells·mL^−1^. The cells were then stained with annexin V–FITC or annexin V–APC and PI dyes for 15 min at room temperature as described by the product protocol, detected through flow cytometry (FITC, peak excitation wavelength [Ex]: 495 nm/peak emission wavelength [Em]: 525 nm; PI, Ex: 535 nm/Em: 615 nm; APC, Ex: 650 nm/Em: 660 nm), and analyzed with WinMDI 2.9 software (Purdue University Cytometry Laboratories, IN, United States).

### Immunofluorescence staining

LX-2 cells cultured on slides were washed with PBS and fixed with 4% polyformaldehyde for 20 min. Then, the cells were permeated with 0.5% Triton-X 100 for 15 min, blocked with 5% BSA in PBS for 30 min at room temperature, and incubated with the Col1A1 primary antibody (Supplementary Table S2) at 4 C for 12 h Cy3-conjugated or FITC-conjugated secondary antibodies (Supplementary Table S2) were used to visualize the primary antibodies, and DAPI or Hoechst 33342 was applied to visualize nuclei. The stained cells were mounted with antifluorescence quenching solution and viewed with a FV10C-W3 laser confocal microscope or a DP80 fluorescence inverted microscope (Olympus, Inc., TKY, Japan).

### RNA isolation, reverse transcription, and qPCR

After treatment, the liver tissues were homogenized, and the LX-2 cells were lysed. Then, the total mRNA was extracted through the Trizol method in accordance with the manufacturer’s instructions. Reverse transcription and PCR were conducted as previously described (Liu et al., 2020). Primers (Supplementary Table S3) were designed and synthesized by Takara Bio (Takara Biotechnology Inc., Dalian, China).

### Western blot analysis

Tissue homogenates or cell lysates were prepared and Western blot analysis was performed in accordance with previously described methods (Zhang et al., 2021). The gray value was analyzed, and the relative expression level of proteins was obtained by using ImageJ 1.51 software (NIH Image, Bethesda, MD, United States). The primary and secondary antibodies were purchased from Abcam (Abcam Inc., Shanghai, China) and are listed in Supplementary Table S2.

### Statistical analysis

All the experiments were performed three times, and data were expressed as mean ± standard deviation. Student’s *t* test was used to compare the differences between two groups, whereas those among three and more groups were compared by using one-way analysis of variance with Tukey’s posthoc test. All statistical tests were two-sided, and *p* < 0.05 was considered as statistically significant.

## Results

### SNS-032 improves liver function and fibrotic stage *in vivo*

**FIGURE 1:**
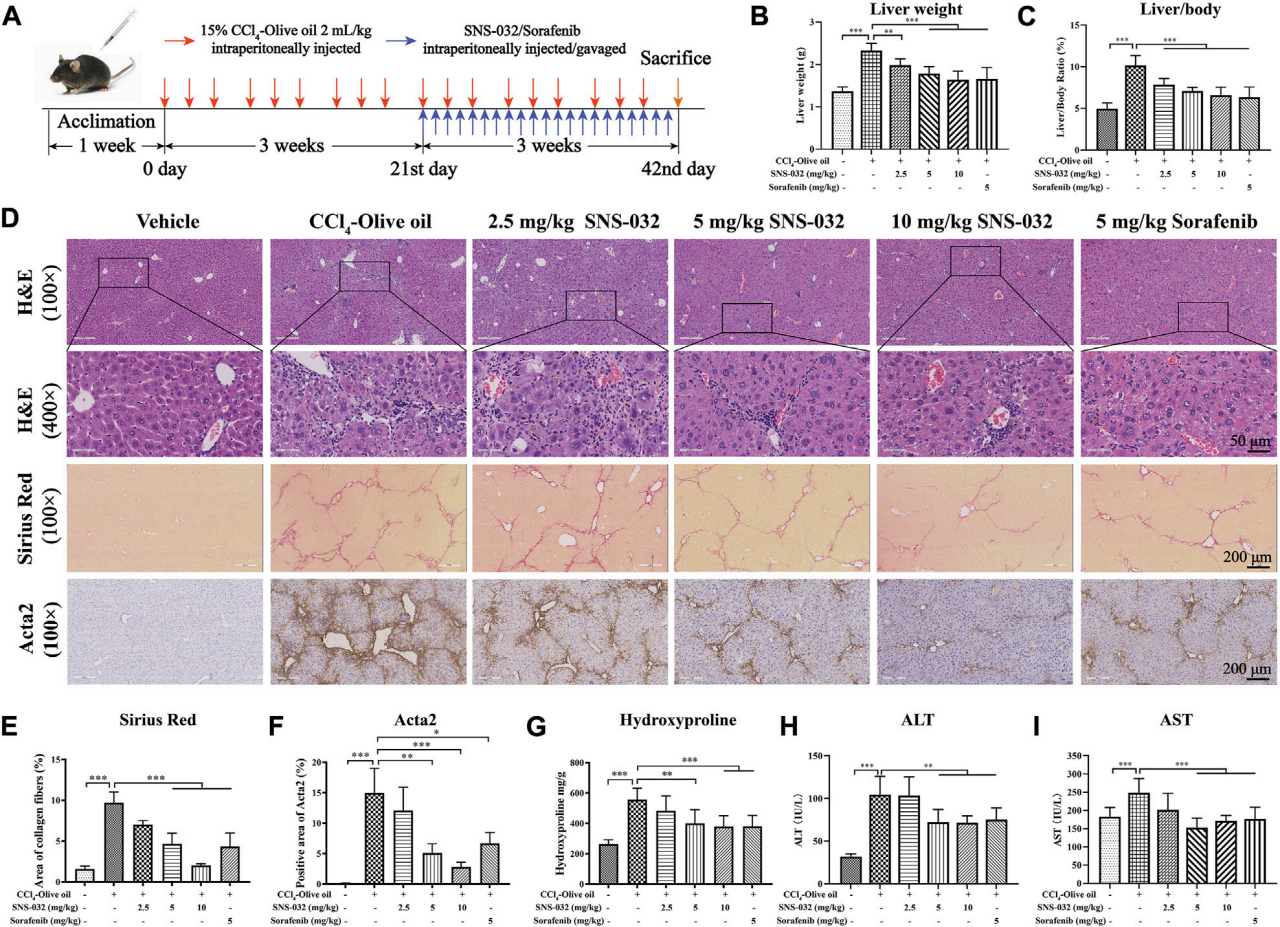
SNS-032 improves hepatic injury and liver fibrosis in CCl_4_-induced mice. Male C57BL/6 mice were intraperitoneally injected with 2 ml/kg 15% CCl_4_-olive oil on Monday, Wednesday and Friday for 6 weeks to establish a hepatic fibrosis model and treated with 2.5, 5, and 10 mg·kg^−1^ SNS-032 or 5 mg·kg^−1^ sorafenib for 3 weeks. Each group had 8 samples (*n* = 8). **(A)** The flowchart of murine model establishment and treatment. **(B)** Liver weight. **(C)** Liver/body ratio. **(D)** H&E staining (magnification: ×100 and ×400), Sirius Red staining (magnification: ×100), and immunohistochemical staining of Acta2 (magnification: ×100) were performed to observe the histopathological characteristics of liver tissue after the treatment of SNS-032 or sorafenib. Then, the semi-quantitative of Sirius Red staining **(E)**, and Acta2 staining **(F)**, were calculated. And the content of hydroxylysine in liver tissue **(G)**, serum ALT **(H)**, and AST **(I)**, were detect. Comparing with experimental group, ^*^ *p* < 0.05, ^**^ *p* < 0.01, ^***^ *p* < 0.001.

Male C57BL/6 mice were intraperitoneally injected with 2 ml·kg^−1^ 15% CCl_4_–olive oil on Mondays, Wednesdays, and Fridays for 6 weeks to establish a murine model of hepatic fibrosis. After 3 weeks of injection, the mice were treated with 2.5, 5, or 10 mg·kg^−1^ SNS-032 and 5 mg·kg^−1^ sorafenib for 3 weeks (Figure 1A). Then, the sera and liver tissues of each group were collected. We found that the liver weight and liver/body weight ratio of the mice in the experimental group had significantly increased compared with those of the mice in the normal group (*p* < 0.001). Liver weight and liver/body weight ratio prominently decreased after treatment with 2.5, 5, or 10 mg·kg^−1^ SNS-032 and 5 mg·kg^−1^ sorafenib (*p* < 0.05; *p* < 0.001; *p* < 0.001) (Figures 1B,C).

HE and Sirius red staining revealed that the deposition of fibers and collagen, the infiltration of inflammatory cells, and the degree of liver fibrosis had increased in the experimental group. We found that after treatment with 5 and 10 mg·kg^−1^ SNS-032, collagen deposition, inflammatory cell infiltration, and liver fibrosis degree significantly decreased (Figure 1D). The semiquantitatively analyzed Sirius red positive area and hydroxyproline content in liver tissue significantly increased in the experimental group but decreased in the 5 and 10 mg·kg^−1^ SNS-032 and 5 mg·kg^−1^ sorafenib groups (*p* < 0.05; *p* < 0.001; *p* < 0.001) (Figures 1D,E,G). The percentage of the Acta2-positive area significantly increased in the experimental group but decreased in the 5 and 10 mg·kg^−1^ SNS-032 and 5 mg·kg^−1^ sorafenib groups (*p* < 0.01; *p* < 0.001; *p* < 0.05) (Figures 1D,F). In terms of biochemical profiles, the serum levels of ALT and AST were significantly elevated in the experimental group and significantly decreased in the 5 and 10 mg·kg^−1^ SNS-032 and 5 mg·kg^−1^ sorafenib groups (*p* < 0.05; *p* < 0.001; *p* < 0.001) (Figures 1H,I).

**FIGURE 2:**
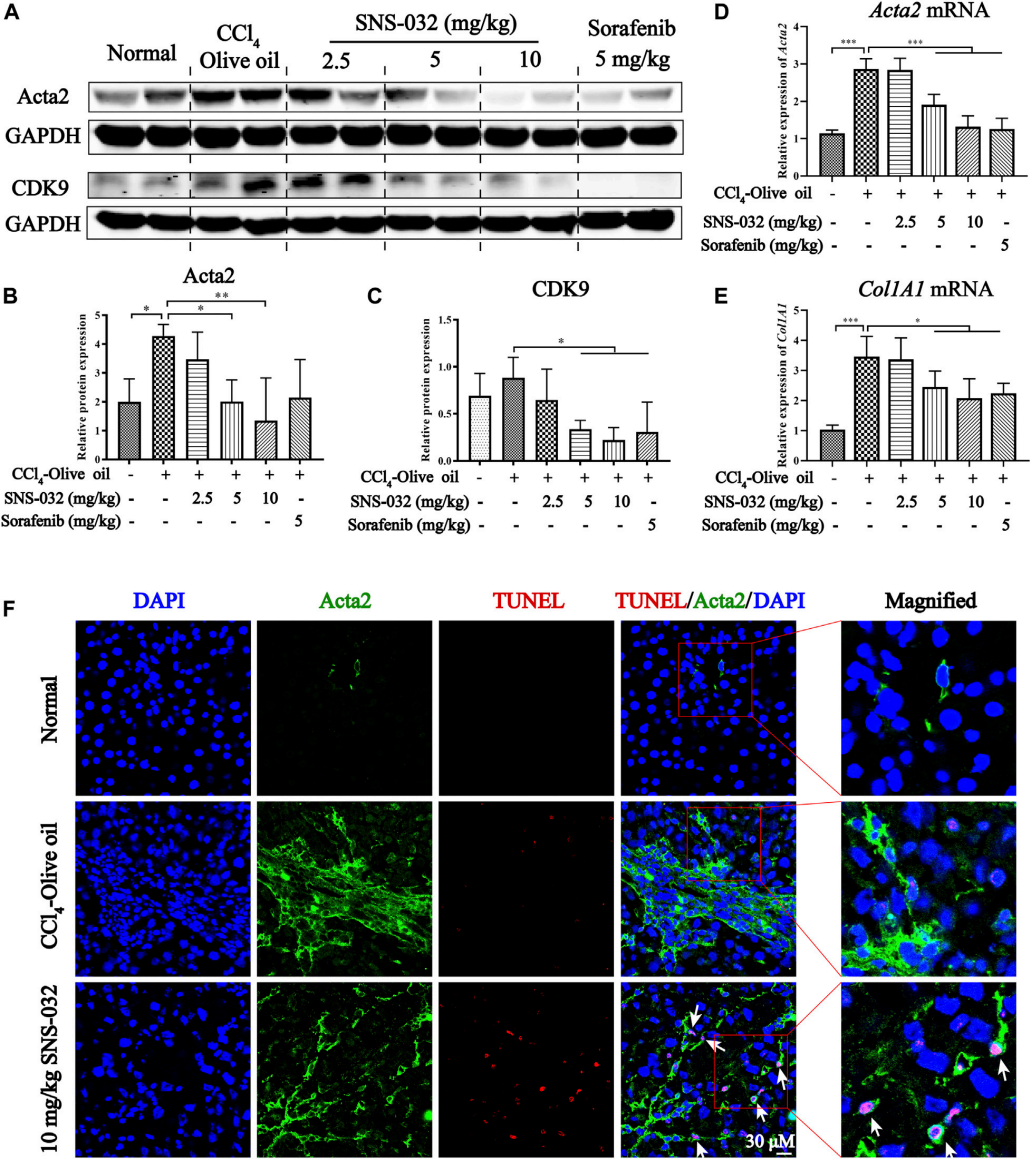
SNS-032 alleviates liver fibrosis and induces the apoptosis of active HSCs *in vivo*. Male C57BL/6 mice were intraperitoneally injected with 2 ml/kg 15% CCl_4_-olive oil on Monday, Wednesday and Friday for 6 weeks to establish a hepatic fibrosis model and treated with 2.5, 5, and 10 mg·kg^−1^ SNS-032 or 5 mg·kg^−1^ sorafenib for 3 weeks **(A)** The protein expressions of Acta2 and CDK9. **(B**,**C)** The relative grey values of Acta2 and CDK9. **(D**,**E)** The mRNA expressions of *Acta2* and *Col1A1*. **(F)** The co-immunofluorescence staining of Acta2, TUNEL, and DAPI in liver tissue (magnification: ×600). Comparing with experimental group, ^*^ *p* < 0.05, ^**^ *p* < 0.01, ^***^ *p* < 0.001.

Then, the protein and mRNA expression levels of Acta2 and Col1A1 were assessed in liver tissues. The protein expression of Acta2 significantly increased in the experimental group but decreased in the 5 and 10 mg·kg^−1^ SNS-032 groups (*p* < 0.05; *p* < 0.01) (Figures 2A,B). Compared with that in the experimental group, the protein expression of CDK9 had significantly decreased in the 5 and 10 mg·kg^−1^ SNS-032 and 5 mg·kg^−1^ sorafenib groups (*p* < 0.05; *p* < 0.05; *p* < 0.05) (Figures 2A,C). The mRNA expression levels of *Acta2* (*p* < 0.001; *p* < 0.001; *p* < 0.001) and *Col1A1* (*p* < 0.05; *p* < 0.05; *p* < 0.05) significantly increased in the experimental group but decreased in the 5 and 10 mg·kg^−1^ SNS-032 and 5 mg·kg^−1^ sorafenib groups (Figures 2D,E). Compared with that in the experimental group, the protein expression of CDK9 in the liver tissues in the 10 mg·kg^−1^ SNS-032 group had significantly decreased (Figures 2A,C). Interestingly, we found that active HSCs expressing Acta2 (green) underwent significant apoptosis after treatment with 10 mg·kg^−1^ SNS-032 (Figure 2F). These results illustrated that SNS-032 improves liver function and ameliorates hepatic inflammation and fibrosis in fibrotic model mice. Moreover, it induces the apoptosis of active HSCs *in vivo*. This effect may be related to the inhibition of CDK9.

### SNS-032 induces the apoptosis of active HSCs and inhibits the proliferation and activation of HSCs *in vitro*

**FIGURE 3:**
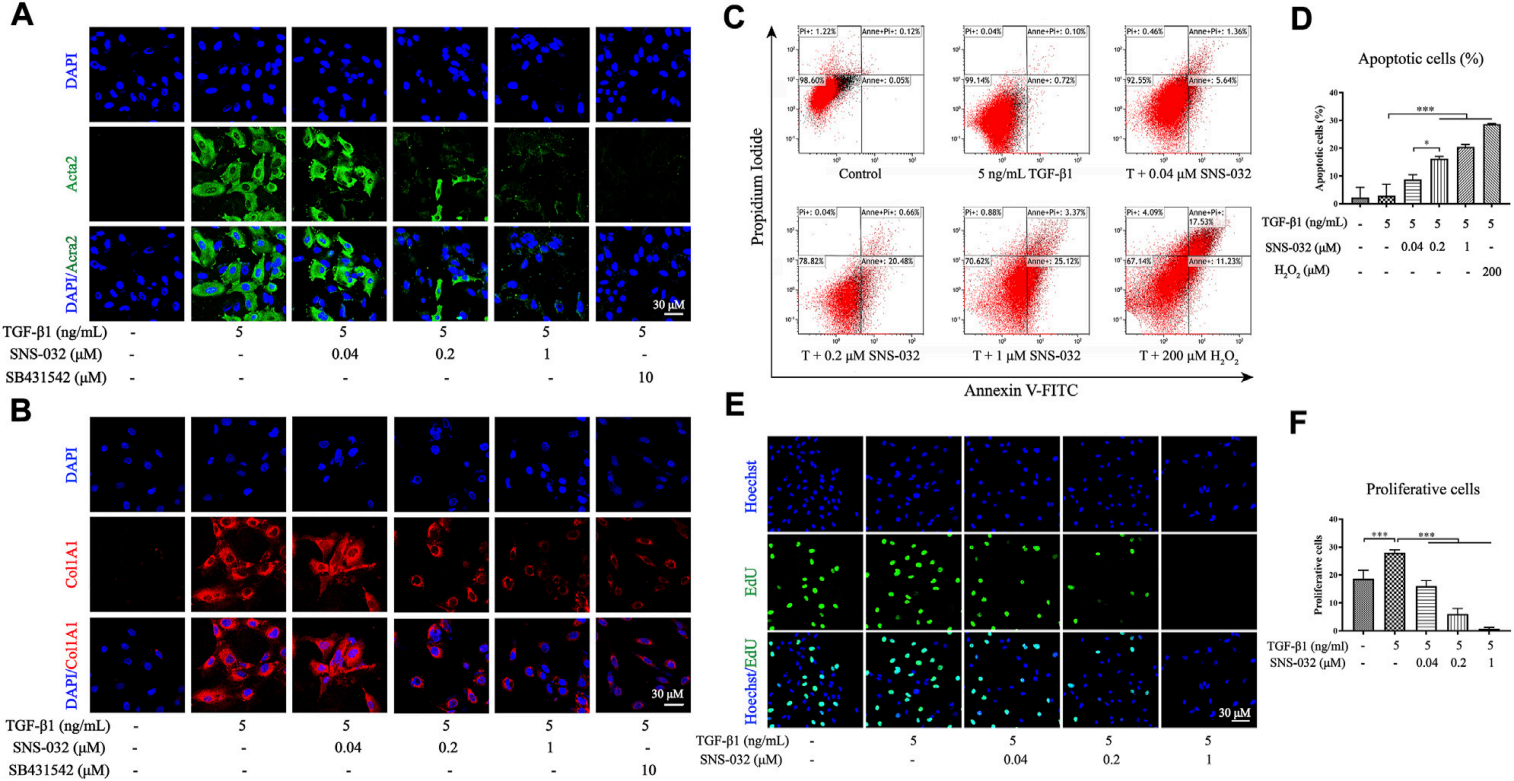
SNS-032 inhibits the activation and proliferation of LX-2 induced by TGF-β1, and promotes its apoptosis. LX-2 cells were incubated with 5 ng·mL^−1^ TGF-β1 and treated with 0.04, 0.2, 1 μM SNS-032, and 10 μM SB431542 (TGF-β1 inhibitor) for 24 h. The immunofluorescence staining of Acta2 (**A**, red) and Col1A1 (**B**, red) were observed by laser confocal microscope (magnification: ×600). Then, LX-2 cells were incubated with 5 ng mL^−1^ TGF-β1 and treated with 0.04, 0.2, 1 μM SNS-032 for 24 h, and 200 μM H_2_O_2_ for 6 h. The apoptotic cells of active LX-2 were stained with Annexin V-FITC/PI/PI and analyzed by flow cytometry **(C)**. **(D)** The percentage of apoptotic cells. **(E)** The proliferation of active LX-2 detected by EdU (green) staining in LX-2 induced by 5 ng·mL^−1^ TGF-β1 (magnification: ×300). **(F)** The numbers of proliferating cells in each high power field. Magnification: ×300. Comparing with experimental group, ^*^ *p* < 0.05, ^**^ *p* < 0.01, ^***^ *p* < 0.001.

*In vitro*, LX-2 cells were activated through incubation with 5 ng·mL^−1^ recombinant human TGF-β1 for 24 h as previously described (He et al., 2020). Acta2, a biomarker of active HSCs, and Col1A1 in TGF-β1-induced LX-2 were detected *via* immunofluorescence staining, and 10 μM SB431542 was used as the positive drug. The results showed that the expression levels of Acta2 and Col1A1 significantly increased in the experimental group but decreased in the 0.2 and 1 μM SNS-032 groups (Figures 3A,B). Then, the active LX-2 cells were treated with different concentrations of SNS-032 and 200 μM H_2_O_2_ for 24 h. Apoptotic cells were stained with Annexin V–FITC/PI dye and detected through flow cytometry. Compared with the treatment received by the experimental group, treatment with 0.2 or 1 μM SNS-032 and 200 μM H_2_O_2_ significantly increased the percentage of active LX-2 in the early and late stages of apoptosis (*p* < 0.001; *p* < 0.001; *p* < 0.001) (Figures 3C,D). Then, proliferative cells were marked through EdU (Azide 488, green) staining. Compared with the treatment received by the experimental group, treatment with 0.04, 0.2, or 1 μM SNS-032 significantly decreased the quantities of proliferative active LX-2 (*p* < 0.001; *p* < 0.001; *p* < 0.001) (Figures 3E,F). These results indicated that SNS-032 induced the apoptosis of active HSCs and inhibited the proliferation and activation of HSCs.

### SNS-032 decreases CDK9 and inhibits the phosphorylation of RNAP II and its downstream signal transductors

**FIGURE 4:**
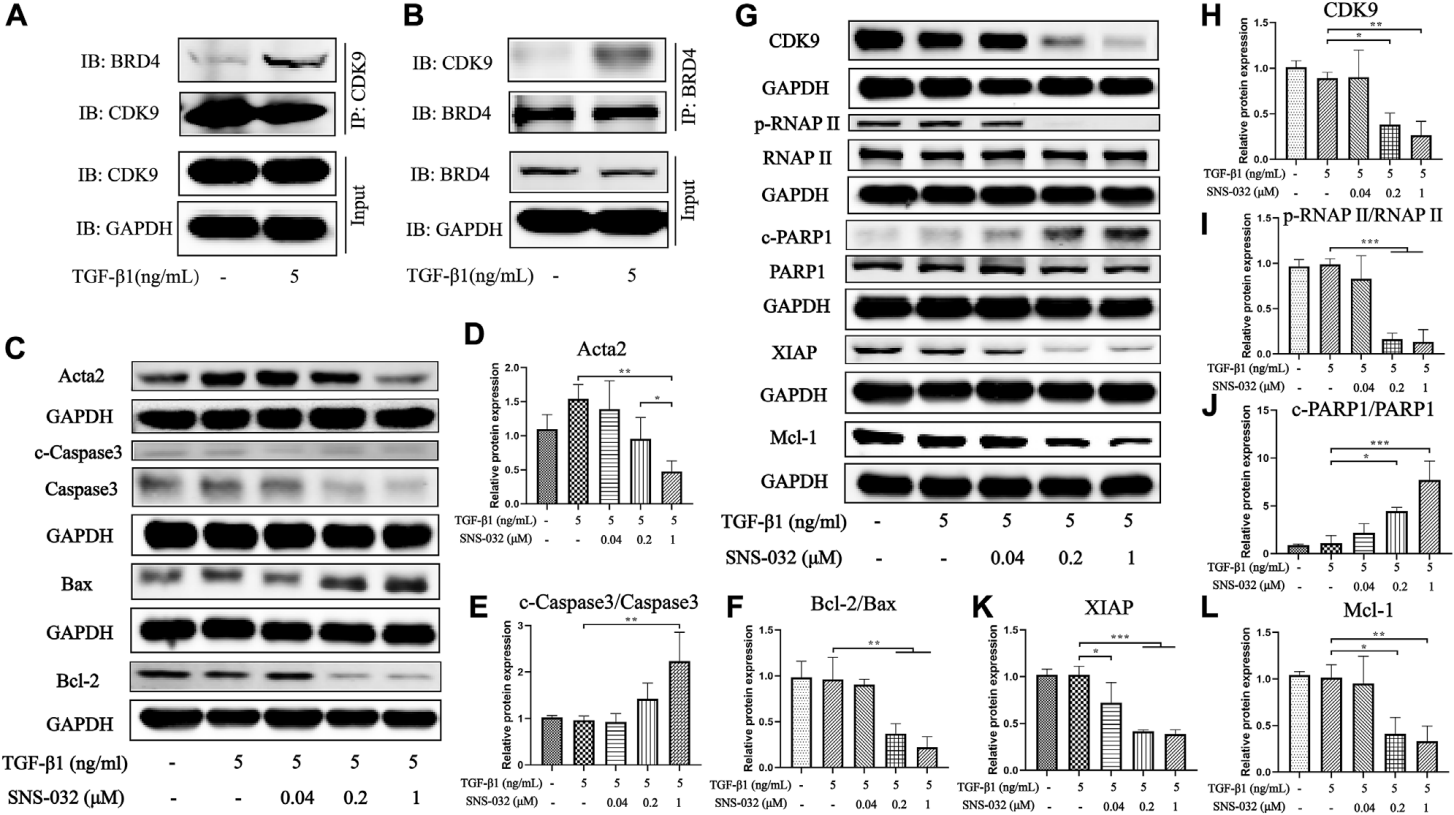
SNS-032 inhibits the expression of CDK9 and its downstream, down-regulates Acta2 and Bcl-2, and up-regulates cleaved-Caspase3 and Bax. LX-2 cells were incubated with 5 ng·mL^−1^ TGF-β1 and treated with 0.04, 0.2, 1 μM SNS-032 for 24 h **(A**,**B)** The immunocoprecipitation (IP) assay between BRD4 and CDK9 in LX-2 cells incubated with 5 ng·mL^−1^ TGF-β1 for 24 h. **(C)** The immunoblot of Acta2 (the activation marker of HSCs), cleaved-Caspase3, Caspase3, Bax, and Bcl-2 (the apoptosis related markers). **(D**–**F)** The relative protein expression of Acta2, cleaved-Caspase3/Caspase3, Bcl-2/Bax. **(G)** The immunoblot of CDK9 and its downstream (phosphorylated RNAP II, RNAP II, cleaved-PARP1, PARP1, XIAP, and Mcl-1). **(H**–**L)** The relative protein expression of CDK9, phosphorylated RNAP II/RNAP II, XIAP, Mcl-1, and cleaved-PARP1/PARP1.

We further investigated the protein expression levels of CDK9 and its downstream factors to clarify the mechanism of SNS-032. First, we compared the expression levels of CDK9 in normal and active LX-2 cells. Interestingly, the protein expression of CDK9 in LX-2 cells was unchanged after treatment with 5 ng·mL^−1^ TGF-β1. According to the literature, CDK9 binds to cyclins (T1, T2a, T2b, and K) to form the heterodimer kinase P-TEFb (Fujinaga, 2020). Usually, P-TEFb is inactive when it is bound to HEXIM1, MePCE, Larp7, and 7SK snRNP (Fujinaga et al., 2014). When cells experience DNA damage as a result of chemical or physical injury, inactive P-TEFb releases HEXIM1, MePCE, Larp7, and 7SK snRNP and recruits BRD4 to form a catalytically active complex that promotes the phosphorylation of RNAP II (Zheng et al., 2021). Therefore, we investigated the combination of CDK9 and BRD4 in active LX-2 cells and found that the conjugation of CDK9 and BRD4 had increased significantly in active LX-2 cells relative to that in normal LX-2 cells (Figures 4A,B). As the CDK9–cyclin–BRD4 complexes increased, the activity of RNAP II significantly increased.

Then, the expression of CDK9 significantly decreased after treatment with 0.2 and 1 μM SNS-032 (*p* < 0.05; *p* < 0.01) (Figures 4G,H). Treatment with 0.2 and 1 μM SNS-032 significantly blocked the phosphorylation of the carboxyterminal domain of RNAP II at Ser2 in the initial stage (*p* < 0.001; *p* < 0.001) (Figures 4G,I). Recent studies have reported that phosphorylated RNAP II promotes the elongation of mRNA and regulates several apoptosis- and proliferation-related proteins, such as XIAP, Mcl-1, and PARP1 (Maccallum et al., 2005). In this study, treatment with 0.2 and 1 μM SNS-032 prominently increased the ratio of cleaved PARP1/PARP1 (*p* < 0.05; *p* < 0.001), and decreased the ratio of Bcl-2/Bax (*p* < 0.01; *p* < 0.01) and the expression levels of XIAP (*p* < 0.001; *p* < 0.001) and Mcl-1 (*p* < 0.05; *p* < 0.01) (Figures 4C,F,G,J–L). Treatment with 1 μM SNS-032 significantly elevated the ratio of cleaved-Caspase3/Caspase3 (*p* < 0.01), and reduced the expression of Acta2 (*p* < 0.01) (Figures 4C–E). These results illustrated that SNS-032 induced the apoptosis of active HSCs and inhibited the proliferation and activation of HSCs by downregulating CDK9, p-RNAP II, XIAP, Mcl-1, Bcl-2, and Acta2 and upregulating Bax, cleaved PARP1, and cleaved Caspase3.

### CDK9 knockdown induces apoptosis and inhibits the proliferation and activation of active LX-2

**FIGURE 5:**
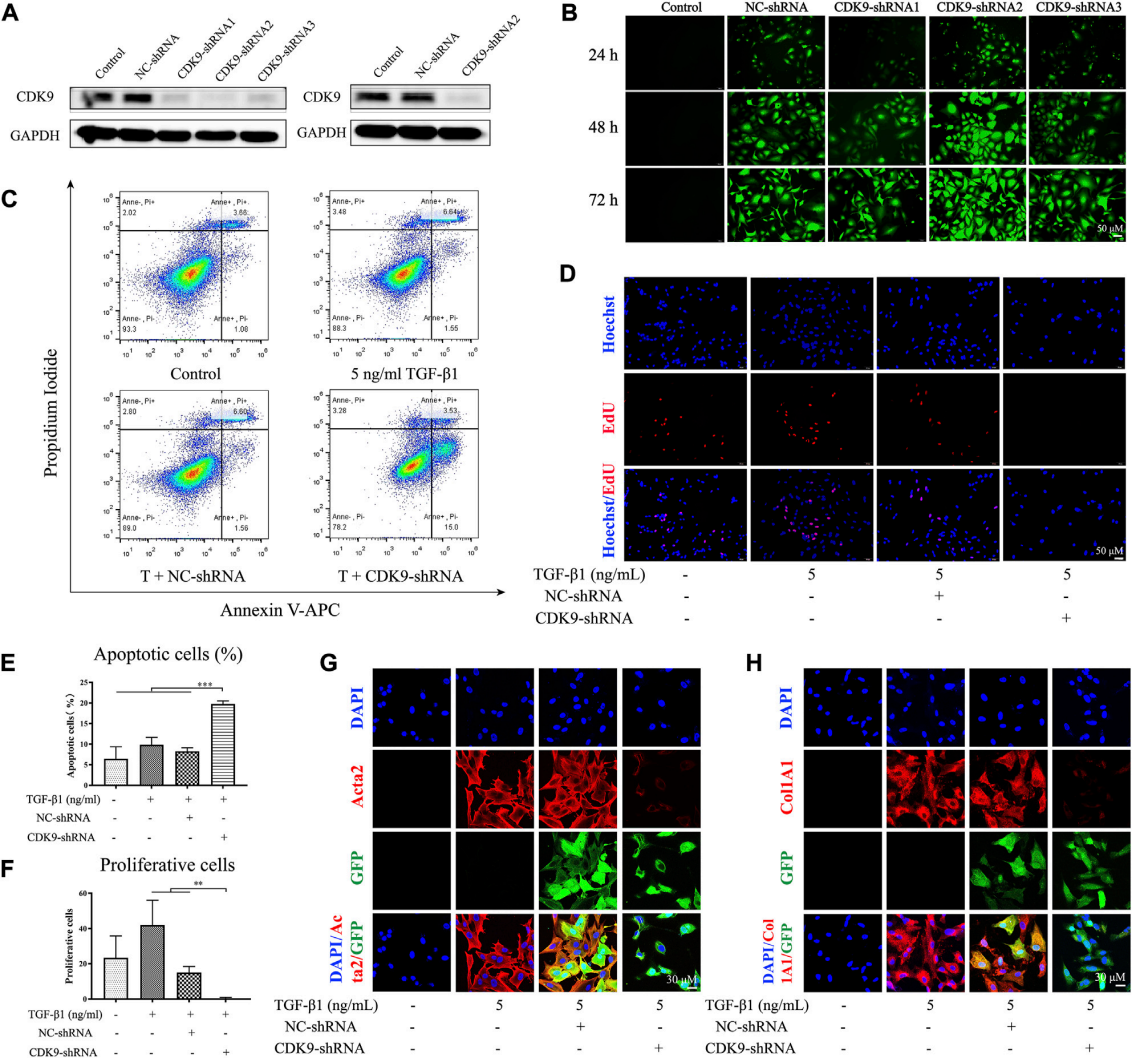
Knocking down CDK9 induces the apoptosis of active LX-2 and inhibits its activation and proliferation. LX-2 were transfected with CDK9-shRNA vectors which also encoded GFP and puromycin resistance genes. The most efficient CDK-shRNA vector was used to transfect LX-2 cells and the protein expression of CDK9 was detected by immunoblot **(A)**. **(B)** The immunofluorescence of GFP (green) after the transfection of CDK9-shRNA for 24, 48, and 72 h. Then, CDK9-shRNA transfected LX-2 cells were incubated with 5 ng·mL^−1^ TGF-β1 for 24 h and the apoptosis of cells were stained with Annexin V-FITC/PI and detected by flow cytometry **(C)**. **(D)** CDK9-shRNA transfected LX-2 cells were stained with EdU (red) and observed by fluorescence inverted microscope (magnification: ×300). **(E)** The percentage of apoptotic cells. **(F)** The numbers of proliferating cells in each high power field. After incubated with 5 ng·mL^−1^ TGF-β1, CDK9-shRNA transfected LX-2 cells were stained with Acta2 **(G)** and Col1A1 **(H)** (magnification: ×600). Acta2 (red), Col1A1 (red), GFP (green), and DAPI (blue). Comparing with experimental group, ^*^ *p* < 0.05, ^**^ *p* < 0.01, ^***^ *p* < 0.001.

Although SNS-032 is a CDK9 inhibitor and shows good effects in liver fibrosis treatment, the mechanisms of CDK9 in the development of liver fibrosis remain unclear. Therefore, we utilized a CDK9-shRNA-loaded lentivirus (GV248) to knockdown CDK9 in LX-2. Three CDK9 shRNAs encoded with base sequences were transfected into LX-2 cells (the three sequences of the CDK9 shRNAs are listed in Supplementary Table S1). Then, the expression of CDK9 and the fluorescence intensity of GFP were detected. Given that CDK9 shRNA2 showed the best transfection and knockdown effects among the three shRNAs (Figures 5A,B), it was used for transfection in further experiments. After transfection with CDK9 shRNA, LX-2 was treated with 4 ng·mL^−1^ puromycin to select stably transfected LX-2 cells (Figures 5A,B). The stably transfected LX-2 cells were cultured with 5 ng·mL^−1^ TGF-β1 in six-well dishes for 24 h, stained with Annexin V–APC/PI, and subjected to flow cytometry. Compared with the negative control (NC shRNA) group, the CDK9 shRNA group showed a higher percentage of apoptosis (*p* < 0.001) (Figures 5C,E). Then, the CDK9 shRNA LX-2 cells were cultured with 5 ng·mL^−1^ TGF-β1 in 48-well dishes for 24 h, stained with EdU (Azide 594, red), and observed by using a laser confocal microscope. Compared with those in the NC shRNA group, the proliferative cells in the CDK9 shRNA group had decreased significantly (*p* < 0.01) (Figures 5D,F). Subsequently, CDK9 shRNA LX-2 cells were cultured with 5 ng·mL^−1^ TGF-β1 in 6 cm dishes for 24 h, incubated with Acta2 and Col1A1 primary antibodies, stained with Cy3 and DAPI, and observed by using a laser confocal microscope. Compared with those in the NC shRNA group, the expression levels of Acta2 and Col1A1 had decreased significantly in the CDK9 shRNA group (Figures 5G,H). Therefore, the knockdown of CDK9 induces the apoptosis of active LX-2 and inhibits the proliferation and activation of active LX-2.

### Knocking down CDK9 decreases CDK9 and inhibits the phosphorylation of RNAP II and its downstream factors

**FIGURE 6:**
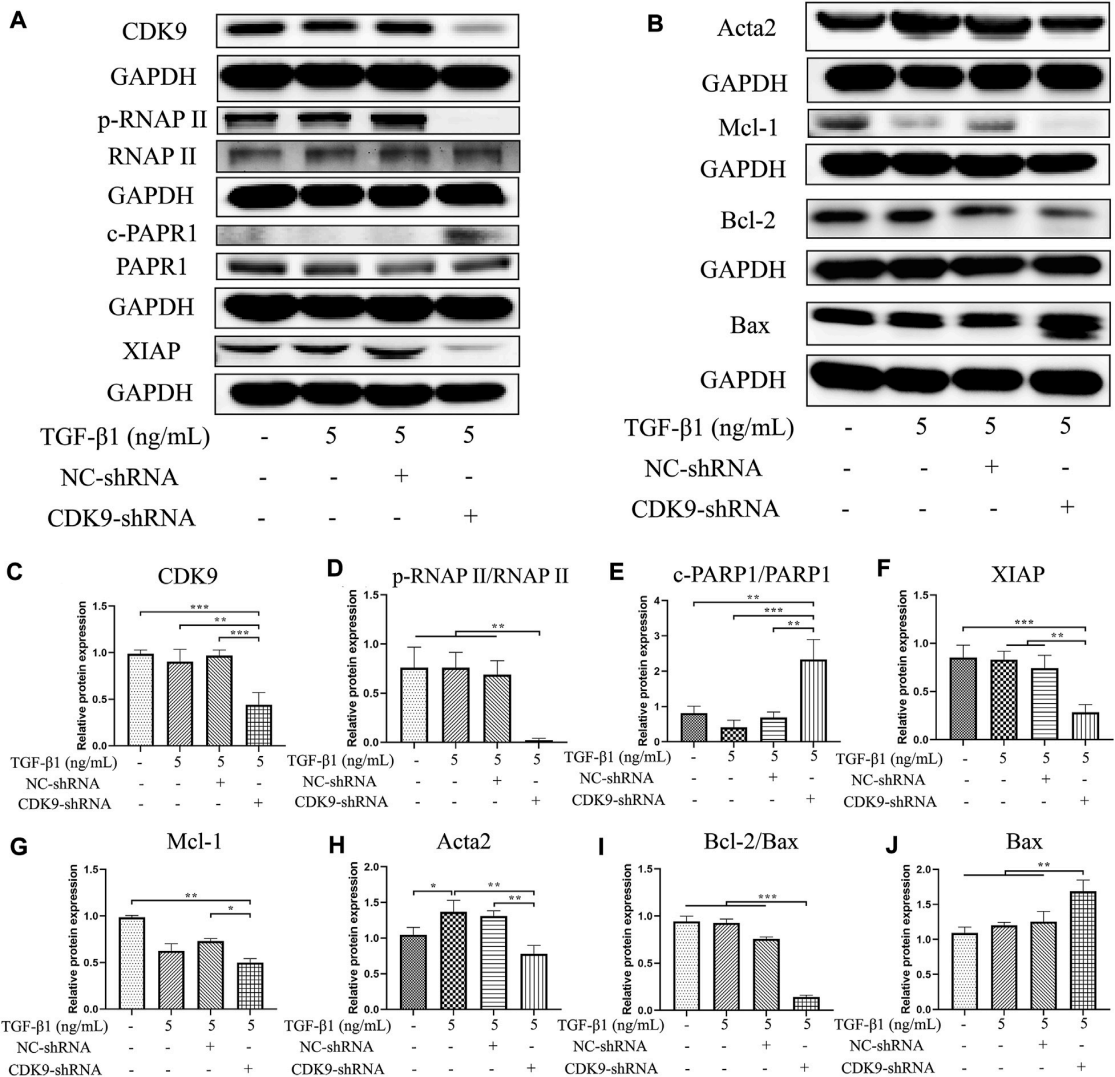
Knocking down CDK9 inhibits the expression of CDK9 and its downstream, down-regulates Acta2 and Bcl-2, and up-regulates Bax. CDK9-shRNA transfected LX-2 cells were incubated with 5 ng·mL^−1^ TGF-β1 for 24 h and the protein expressions of CDK9 and its downstream were detected by immunoblot. **(A**,**B)** The immunoblot of CDK9, its downstream (phosphorylated RNAP II, RNAP II, cleaved-PARP1, PARP1, XIAP, and Mcl-1), Acta2 (the activation marker of HSCs), Bcl-2, and Bax (the apoptosis related markers). **(C**–**J)** The relative protein expressions of CDK9, phosphorylated RNAP II/RNAP II, cleaved-PARP1/PARP1, XIAP, Mcl-1, Acta2, Bcl-2/Bax, and Bax. Comparing with experimental group, ^*^ *p* < 0.05, ^**^ *p* < 0.01, ^***^ *p* < 0.001.

Compared with that in the NC shRNA group, the expression of CDK9 significantly decreased in the group with CDK9 gene knockdown (*p* < 0.001) (Figures 6A,C). Knockdown of CDK9 significantly blocked the phosphorylation of the carboxyterminal domain of RNAP II at Ser2 in the initial stage (*p* < 0.01) (Figures 6A,D). Knockdown of CDK9 significantly decreased the ratio of cleaved PARP1/PARP1 increased (*p* < 0.01) and the expression levels of XIAP (*p* < 0.01) and Mcl-1 (*p* < 0.05) (Figures 6A,B,E–G). Knockdown of CDK9 prominently increased the expression of Bax (*p* < 0.01), and reduced the expression of Acta2 (*p* < 0.01) and the ratio of Bcl-2/Bax (*p* < 0.001) (Figures 6B,E–G). Similar to the effects of SNS-032, knockdown of CDK9 induced the apoptosis of active HSCs and inhibited the proliferation and activation of HSCs by downregulating CDK9, p-RNAP II, XIAP, Mcl-1, Bcl-2, and Acta2 and upregulating Bax and cleaved PARP1.

## Discussion

CDK9 is a potential target that has widely attracted attention in cancer studies. Similar to traditional CDKs, CDK9 plays an important role in the transcription of mRNA and is closely related to the survival and proliferation of cells (Santo et al., 2015). A growing body of evidence suggests that the expression of CDK9 is elevated in malignant cells, such as chronic lymphocytic leukemia, multiple myeloma, breast cancer, and lung cancer cells (Franco et al., 2018). Liver fibrosis is a pathological process that occurs along with the combination of damage and repair. Excessive ECM accumulation results in fiber and collagen deposition in hepatic sinuses; this effect blocks the regeneration of liver tissue (Parola and Pinzani, 2019). ECM is secreted by active HSCs, which are activated and proliferate vigorously when liver injury occurs (Reichert et al., 2021). Therefore, antiactive HSCs are the key points in the treatment of liver fibrosis. Given that mRNA synthesis can be promoted when cell division or DNA repair occurs, actively proliferating cells exhibit active mRNA synthesis with the phosphorylation of RNAP II (Jia et al., 2021).

**FIGURE 7:**
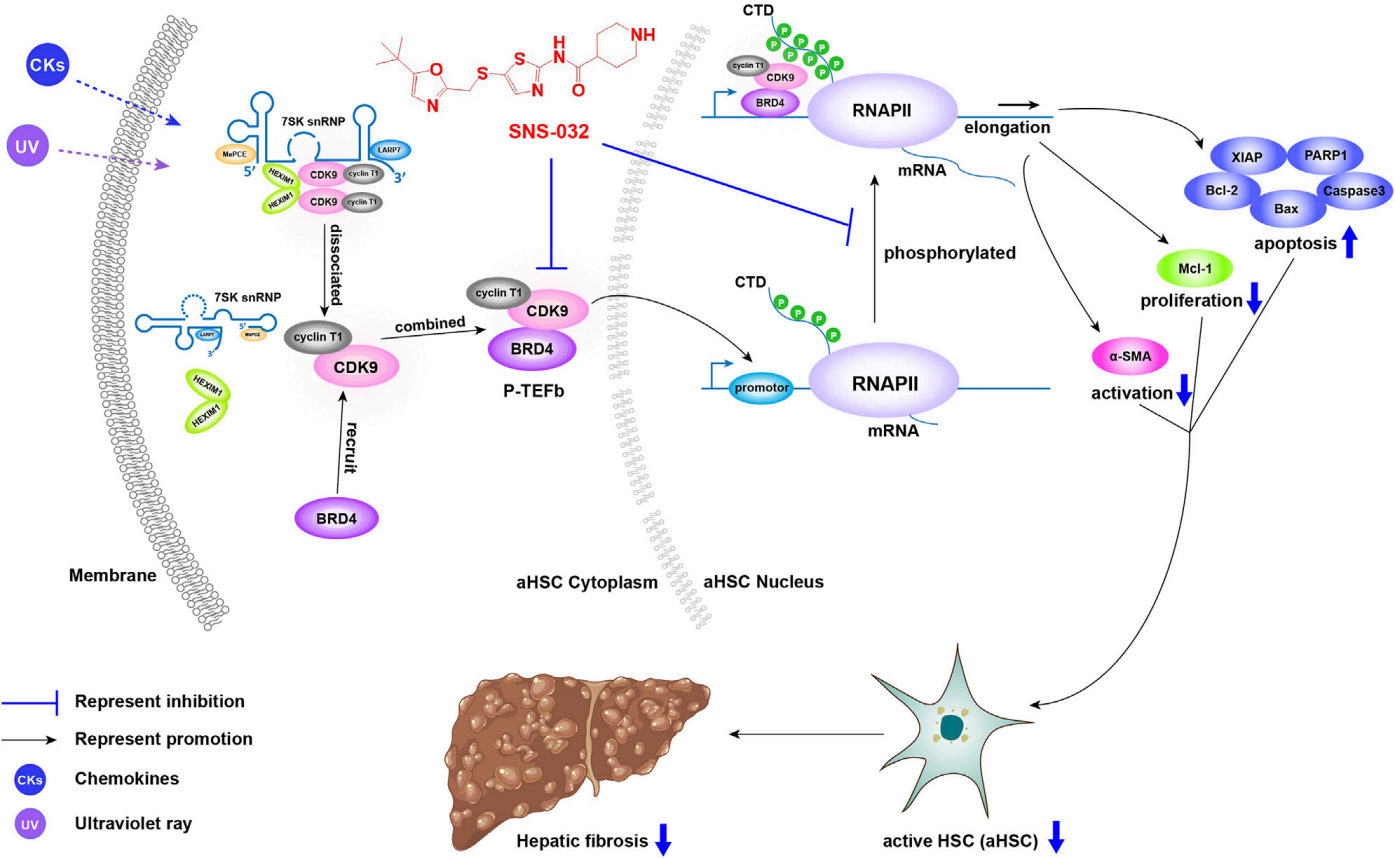
The illustration of the mechanism of CDK9 to promote liver fibrosis and the inhibitory effect of SNS-032. Under physical or chemical irritation, inactive P-TEFb (CDK9–cyclin–HEXIM1–MePCE–Larp7–7SK snRNP complex) releases HEXIM1, MePCE, Larp7, and 7SK snRNP and recruits BRD4 to form active P-TEFb, which catalyzes the phosphorylation of RNAP II. Then, phosphorylated RNAP II upregulates the transcription of anti-apoptosis, proliferation and activation related genes, and downregulates the transcription of apoptosis related genes. SNS-032 inhibits the expression of CDK9, block the phosphorylation of RNAP II and its downstream. It decreases the active HSCs to alleviate the hepatic fibrosis *via* inducing the apoptosis of active HSCs and inhibiting its activation and proliferation.

Recent studies have reported that CDK inhibitors have antiliver fibrosis effects. Roscovitine (a Cdc2, CDK2, and CDK5 inhibitor) protects against liver diseases by inhibiting macrophage inflammatory actions and HSC activation at the onset of liver injury (Liu et al., 2021). CR8, a pan-CDK inhibitor and a derivative of roscovitine, reduces the proliferation and viability of LX-2 cells and attenuates the profibrotic properties of primary murine HSCs while preserving the proliferation and viability of primary murine hepatocytes *in vitro* (Hübbers et al., 2020). In this study, we found that SNS-032 prominently alleviated hepatic fibrosis in CCl_4_-induced model mice. In the experimental group, the expression of CDK9 significantly increased after CCl_4_ induction. Then, after SNS-032 treatment, the expression levels of CDK9 and Acta2 decreased significantly and the apoptosis of active HSCs increased. Notably, CDK9 expression in active and quiescent HSCs *in vitro* did not significantly vary. Similar results were also observed in TGF-β1-induced primary mouse fibroblasts (Qu et al., 2015). Therefore, we further detected the expression of active P-TEFb, which contains CDK9 and BRD4, and found that although the expression of CDK9 was unchanged, the conjugation of CDK9 and BRD4 had increased. Generally speaking, under physical or chemical irritation, inactive P-TEFb (CDK9–cyclin–HEXIM1–MePCE–Larp7–7SK snRNP complex) releases HEXIM1, MePCE, Larp7, and 7SK snRNP and recruits BRD4 for combination into active P-TEFb, which catalyzes the phosphorylation of RNAP II (Zheng et al., 2021). The main function of phosphorylated RNAP II in eukaryotic cells is the transcription elongation of nascent mRNA strands; this process is quite active during cell proliferation (Baluapuri et al., 2019). SNS-032 decreases active HSCs to alleviate hepatic fibrosis by inducing the apoptosis of active HSCs and inhibiting their activation and proliferation by inhibiting the expression of CDK9 and blocking the phosphorylation of RNAP II and its downstream factors (Figure 7).

In this study, we found that SNS-032 significantly downregulated the expression levels of Bcl-2, XIAP, and Mcl-1 (Figure 4). However, some previous studies on other cell types found that SNS-032 has no major effects on the protein levels of XIAP and Bcl-2 (Zhang et al., 2019; Zeng et al., 2021). In our opinion, given that different cell lines undergo different transformations, the regulatory effects of SNS-032 may vary across different cell lines. To illustrate, Chen et al. found that the expression levels of Mcl-1 and XIAP decreased in chronic lymphocytic leukemia cells after 24 h of treatment with SNS-032. Bcl-2 exhibited significantly decreased mRNA expression but not protein expression (Chen et al., 2009). Meanwhile, some studies suggested that even in the same cell type but in different cell lines, the regulatory effects of SNS-032 differ. For example, under SNS-032 treatment, the protein expression of cleaved PARP1 increased significantly in JeKo.1 but not in Granta 519; these cell lines are both human mantle cell lymphoma cell lines (Chen et al., 2010b). Another recent study also supported the supposition that SNS-032 significantly decreased the protein expression of Bcl-2 in SU-DHL-4, a human diffuse large B-cell lymphoma cell line, but had no effect on SU-DHL-2 (Jiang et al., 2022).

In liver tissue, CDK9 has two isoforms with the molecular weights of 55 and 42 kDa (Liu and Herrmann, 2005). Although these isoforms have the same 42 kDa amino acid sequence, CDK9_55_ has an additional 13 kDa amino acid chain (117 residue terminal extension), and its three-dimensional structure differs from that of CDK9_42_. Moreover, they are encoded by the same genes but are regulated by different promoter regions (Shore et al., 2005; Liu et al., 2010). The predominant isoform of CDK9 differs during different stages of the cell cycle. Specifically, CDK9_42_ predominates after the cell cycle, whereas CDK9_55_ predominates before the cell cycle. CDK9_42_ and CDK9_55_ have different locations: CDK9_42_ is located in the nucleoplasm, whereas CDK9_55_ is located in the nucleus (Shore et al., 2003). The specific physiological functions of CDK9_55_ and CDK9_42_ remain unclear. We noticed that the expression of CDK9 varied considerably in the same group, whereas the expression of CDK9_55_ was consistent with that of CDK9_42_ in the same sample (Supplementary Material, uncropped WB figures). In addition, CDK9_42_ is predominant in HSCs, whereas CDK9_55_ is predominant in hepatocytes (Shore et al., 2003). CDK9_42_ inhibitors, if selectively targeting CDK9_42_, may selectively affect HSCs in addition to hepatocytes. This characteristic may help the development of highly selective inhibitors for liver fibrosis treatment.

CDK9 is not only closely related to proliferation and survival, it also regulates activation, growth, and inflammation directly. CDK9 could phosphorylate the Smad linker to drive Smad transcriptional activation and turnover through the BMP and TGF-β pathways (Alarcón et al., 2009). In a mouse model of unilateral ureteral obstruction, CDK9 could also conjugate with Smad3 and Smad4 to form a complex to promote renal fibrosis (Qu et al., 2015). Recent studies have reported that the transient induction of CDK9 in the early stage of differentiation is critical for myogenesis (Tarhriz et al., 2019). CDK9 could also phosphorylate glucocorticoid receptor-interacting protein-1 to regulate the anti-inflammatory effects of glucocorticoids (Rollins et al., 2017). Given that CDK9 could bind with numerous other proteins to form active complexes, future studies may need to focus on the active complexes formed by CDK9 and the upstream and downstream factors of CDK9 to elucidate the mechanism of CDK9 further.

## Conclusion

The CDK9 inhibitor SNS-032 has definite effects in liver fibrosis treatment *in vivo* and *in vitro*. SNS-032 can induce the apoptosis of active HSCs and inhibit the proliferation and activation of active HSCs to alleviate fibrosis by downregulating the expression of CDK9 and its downstream factors. The knockdown of CDK9 exerts the same antifibrosis effects by regulating the CDK9/RNAP II signaling pathway. These pieces of evidence strongly suggest that CDK9 is a potential powerful target against liver fibrosis.

## Data Availability

The original contributions presented in the study are included in the article/Supplementary Material, further inquiries can be directed to the corresponding authors.

## Supplementary material

Supplementary Figure S1 Graphical abstract.

## Glossary

Acta2: actin alpha 2
ALT: alanine aminotransferase
APC: allophycocyanin
AST: aspartate transaminase
Bax: Bcl-2 associated X
Bcl-2: B-cell lymphoma-2
BRD4: bromodomain-containing protein 4
CCl4: Carbon tetrachloride
CDK9: cyclin-dependent kinase 9
Col1A1: collagen type Iα1
DAPI: diaminyl phenyl indole
DMEM: Dulbecco modified Eagle’s medium
ECM: extracellular matrix
FBS: fetal bovine serum
FITC: fluorescein isothiocyanate
GFP: green fluorescent protein
HE: hematoxylin–eosin
HEXIM1: hexamethylene bis-acetamide-inducible protein 1
HSCs: hepatic stellate cells
Mcl-1: myeloid cell leukemia-1
MePCE: methylphosphate capping enzyme
NC: negative control
PARP1: poly ADP-ribose polymerase 1
PI: propidium iodide
RNAP II: RNA polymerase II
P-TEFb: positive transcription elongation factor b
shRNA: short hairpin RNA
SEC: super elongation complex
TGF-β: transforming growth factor-β
XIAP: X-linked inhibitor of apoptosis protein
7SK snRNP: 7SK small nuclear ribonucleoprotein.
