# Supplementary material for: SNS-032 attenuates liver fibrosis by anti-active hepatic stellate cells via inhibition of cyclin dependent kinase 9
Source: Front Pharmacol. 2022 Oct 12;13:1016552. doi: 10.3389/fphar.2022.1016552 (PMC9597511; doi:10.3389/fphar.2022.1016552)
Supplement: Supplementary file 1 [file DataSheet1.doc]

**Original Research Article**

**SNS-032 attenuates liver fibrosis by anti-active hepatic stellate cells *via* inhibition of cyclin dependent kinase 9**

**Xiaoli He ^a, c^, Jiamei Chen ^a^, Dingqi Zhang ^a^, Hailin Yang ^a^, Linzhang Zhang ^a, b^, Yongping Mu ^a^, Hua Zhang ^a^, Gaofeng Chen ^a^, Wei Liu ^a *^ & Ping Liu ^a, b *^.**

^a^ *Key Laboratory of Liver and Kidney Diseases (Ministry of Education), Institute of Liver Diseases, Shanghai Key Laboratory of Traditional Chinese Clinical Medicine, Shuguang Hospital Affiliated to Shanghai University of Traditional Chinese Medicine, 528 Zhangheng Road, Shanghai, 201203, China*

^b^ *Shanghai Frontiers Science Center of TCM Chemical Biology; Institute of Interdisciplinary Integrative Medicine Research, Shanghai University of Traditional Chinese Medicine, Shanghai, 201203, China*

^c^ *Department of Endocrinology, Yueyang Hospital of Integrated Traditional Chinese and Western Medicine, Shanghai University of Traditional Chinese Medicine, Shanghai, 200437, China*

***Correspondence to:** Professor Ping Liu and Associate Professor Wei Liu, Institute of Liver Diseases, Shuguang Hospital Affiliated to Shanghai University of Traditional Chinese Medicine, Shanghai 201203, China. liuliver@vip.sina.com (Ping Liu); lwhzayl@163.com (Wei Liu).

**Supplementary Table 1**

| **NO.** | **5’** | **STEM** | **Loop** | **STEM** | **3’** |
| --- | --- | --- | --- | --- | --- |
| CDK9-RNAi(24626-1)-a | Ccgg | agGGACATGAAGGCTGCTAAT | CTCGAG | ATTAGCAGCCTTCATGTCCCT | TTTTTg |
| CDK9-RNAi(24626-1)-b | aattcaaaaa | agGGACATGAAGGCTGCTAAT | CTCGAG | ATTAGCAGCCTTCATGTCCCT |  |
| CDK9-RNAi(24627-1)-a | Ccgg | ctACTACATCCACAGAAACAA | CTCGAG | TTGTTTCTGTGGATGTAGTAG | TTTTTg |

**Table S1.** The RNA sequences of the three CDK9 short hairpin RNAs.

**Supplementary Table 2**

|  | Antibodies | Anti-species | Inc. | Dilution | Lot. |
| --- | --- | --- | --- | --- | --- |
| Primary antibodies | Acta2 | Rabbit | Abcam | 1: 1000 | ab5694 |
|  | CDK9 | Mouse | Santa Cruz | 1: 1000 | sc-13130 |
|  | BRD4 | Rabbit | Abcam | 1: 1000 | ab128874 |
|  | p-RNA Pol II | Rabbit | Abcam | 1: 1000 | ab5095 |
|  | RNA Pol II | Rabbit | Abcam | 1: 1000 | ab26721 |
|  | cleaved-PARP1 | Rabbit | Abcam | 1: 1000 | ab32064 |
|  | PARP1 | Rabbit | Abcam | 1: 1000 | ab191217 |
|  | cleaved-Caspase3 | Rabbit | Abcam | 1: 1000 | ab32042 |
|  | Caspase3 | Rabbit | Abcam | 1: 1000 | ab32351 |
|  | Mcl-1 | Rabbit | Abcam | 1: 1000 | ab32087 |
|  | Bcl-2 | Rabbit | Abcam | 1: 1000 | ab32124 |
|  | Bax | Rabbit | Abcam | 1: 1000 | ab32503 |
|  | XIAP | Mouse | Santa Cruz | 1: 1000 | sc-55551 |
|  | GAPDH | Mouse | Proteintech | 1: 5000 | 60004-1-Ig |
|  | GAPDH | Rabbit | Proteintech | 1: 5000 | 10494-1-AP |
| Secondary antibodies | Anti-mouse | Goat | Beyotime | 1: 1000 | A0216 |
|  | Anti-rabbit | Goat | Beyotime | 1: 1000 | A0208 |
|  | FITC-anti-mouse | Goat | Abcam | 1: 3000 | ab6785 |
|  | Cy3-anti-rabbit | Goat | Abcam | 1: 3000 | ab6939 |

**Table S2.** The list of primary antibodies and secondary antibodies.

**Supplementary Table 3**

| Primers | Forward | Reverse |
| --- | --- | --- |
| *Acta2*  (mouse) | AATGGCTCTGGGCTCTGTAA | TCTCTTGCTCTGGGCTTCAT |
| *Col1A1*  (mouse) | TGACTGGAAGAGCGGAGAGT | GACGGCTGAGTAGGGAACAC |
| *Gapdh*  (mouse) | AGGTCGGTGTGAACGGATTTG | GGGGTCGTTGATGGCAACA |

**Table S3.** Real-time PCR primer sequences.
