# Supplementary figures and images for: SNS-032 attenuates liver fibrosis by anti-active hepatic stellate cells via inhibition of cyclin dependent kinase 9
Source: Front Pharmacol. 2022 Oct 12;13:1016552. doi: 10.3389/fphar.2022.1016552 (PMC9597511; doi:10.3389/fphar.2022.1016552)

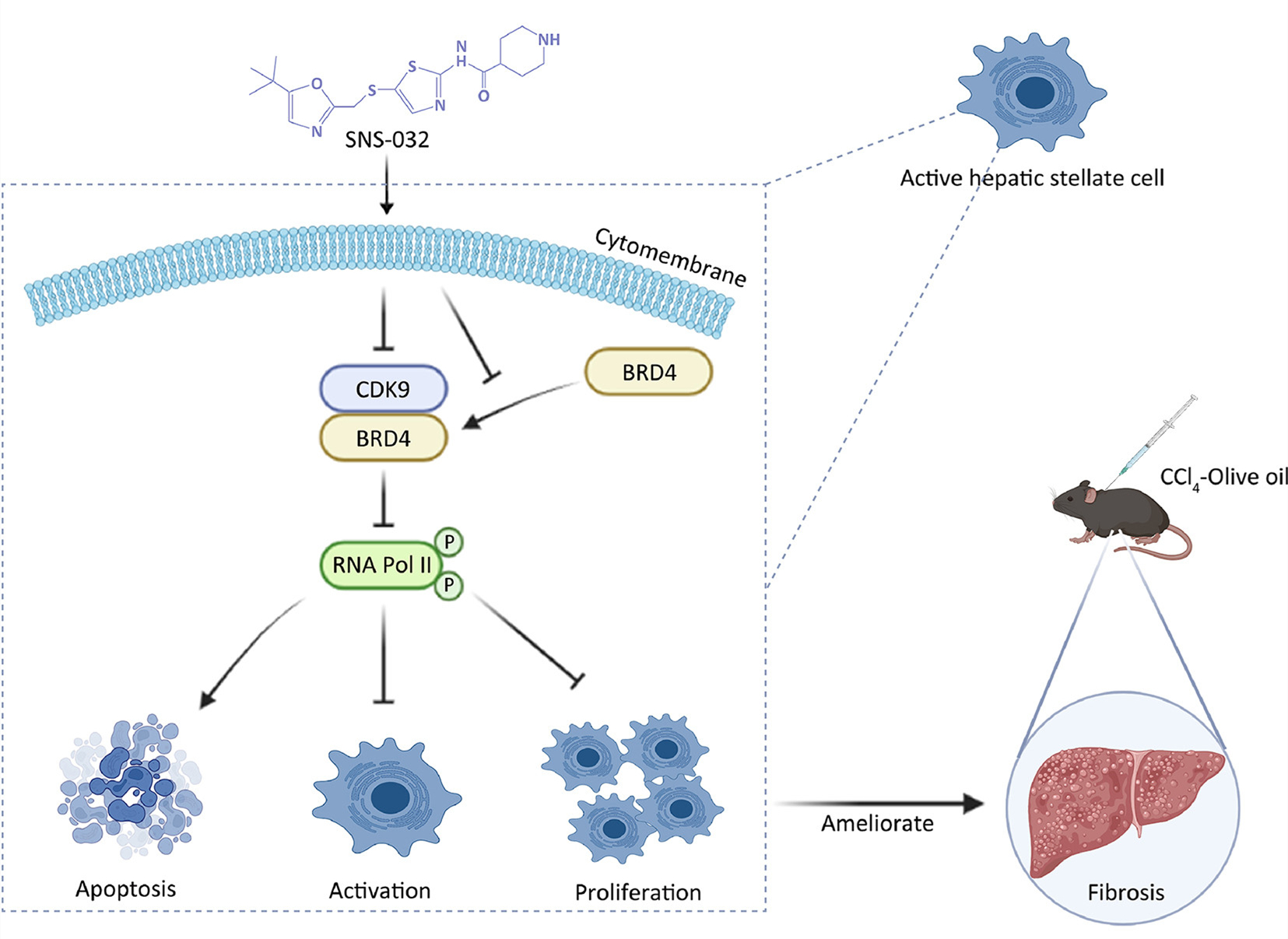

Supplement: Supplementary file 2 [file Image1.JPEG]
